# *N*′-(3,4-Dimethoxy­benzyl­idene)acetohydrazide

**DOI:** 10.1107/S1600536809027366

**Published:** 2009-07-25

**Authors:** Lu-Ping Lv, Tie-Ming Yu, Wen-Bo Yu, Wei-Wei Li, Xian-Chao Hu

**Affiliations:** a Department of Chemical Engineering, Hangzhou Vocational and Technical College, Hangzhou 310018, People’s Republic of China; b Research Center of Analysis and Measurement, Zhejiang University of Technology, Hangzhou 310014, People’s Republic of China

## Abstract

In the title mol­ecule, C_11_H_14_N_2_O_3_, the acetohydrazide group is planar 0.084 (1) Å and forms a dihedral angle of 19.7 (1)° with the benzene ring. One of the meth­oxy groups is coplanar with the attached benzene ring within 0.052 (3) Å, whereas the other is slightly twisted [C—O—C—C = 6.3 (3)°]. The mol­ecule adopts a *trans* configuration with respect to the C=N bond. In the crystal, the mol­ecules are linked into chains along the *a* axis by N—H⋯O hydrogen bonds and the chains are cross-linked into a three-dimensional network by C—H⋯O hydrogen bonds.

## Related literature

For general background to Schiff bases, see: Cimerman *et al.* (1997 ▶); Offe *et al.* (1952 ▶); Richardson *et al.* (1988 ▶). For related structures, see: Li & Jian (2008 ▶); Tamboura *et al.* (2009 ▶).
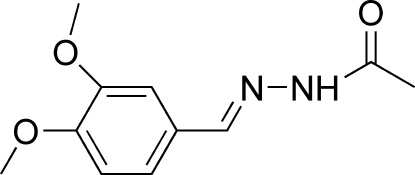


## Experimental

### 

#### Crystal data

C_11_H_14_N_2_O_3_
*M* *_r_* = 222.24
Orthorhombic, 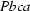

*a* = 8.794 (3) Å
*b* = 10.920 (3) Å
*c* = 24.418 (7) Å
*V* = 2345.0 (12) Å^3^
*Z* = 8
Mo *K*α radiation
μ = 0.09 mm^−1^
*T* = 223 K
0.24 × 0.21 × 0.20 mm

#### Data collection

Bruker SMART CCD area-detector diffractometer
Absorption correction: multi-scan (*SADABS*; Bruker, 2002 ▶) *T*_min_ = 0.977, *T*_max_ = 0.979
11332 measured reflections
2070 independent reflections
1819 reflections with *I* > 2σ(*I*)
*R*_int_ = 0.034

#### Refinement

*R*[*F* ^2^ > 2σ(*F* ^2^)] = 0.050
*wR*(*F* ^2^) = 0.133
*S* = 1.10
2070 reflections
149 parameters
H-atom parameters constrained
Δρ_max_ = 0.15 e Å^−3^
Δρ_min_ = −0.12 e Å^−3^

### 

Data collection: *SMART* (Bruker, 2002 ▶); cell refinement: *SAINT* (Bruker, 2002 ▶); data reduction: *SAINT*; program(s) used to solve structure: *SHELXS97* (Sheldrick, 2008 ▶); program(s) used to refine structure: *SHELXL97* (Sheldrick, 2008 ▶); molecular graphics: *SHELXTL* (Sheldrick, 2008 ▶); software used to prepare material for publication: *SHELXTL*.

## Supplementary Material

Crystal structure: contains datablocks I, global. DOI: 10.1107/S1600536809027366/ci2855sup1.cif

Structure factors: contains datablocks I. DOI: 10.1107/S1600536809027366/ci2855Isup2.hkl

Additional supplementary materials:  crystallographic information; 3D view; checkCIF report

## Figures and Tables

**Table 1:** Hydrogen-bond geometry (Å, °)

*D*—H⋯*A*	*D*—H	H⋯*A*	*D*⋯*A*	*D*—H⋯*A*
N2—H2⋯O3^i^	0.86	1.97	2.803 (2)	164
C1—H1*C*⋯O2^ii^	0.96	2.55	3.435 (3)	153
C11—H11*C*⋯O3^iii^	0.96	2.47	3.425 (3)	174

Symmetry codes: (i) 
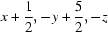
; (ii) 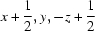; (iii) 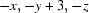.

**Fig. 1.**
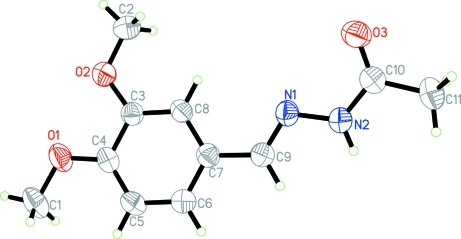
The molecular structure of the title compound. Displacement ellipsoids are drawn at the 40% probability level.

**Fig. 2.**
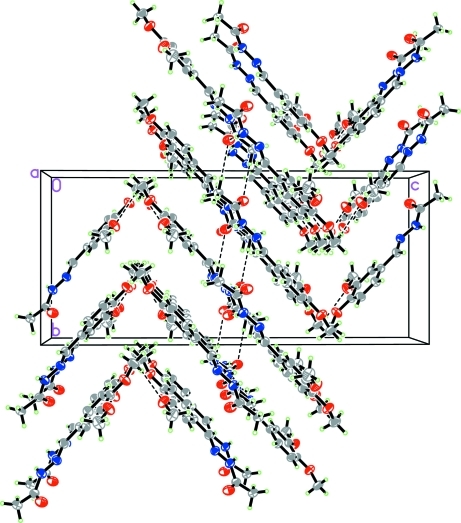
Part of the crystal packing of the title compound. Hydrogen bonds are shown as dashed lines.

## supplementary crystallographic information

### Comment

Schiff bases have attracted much attention due to the possibility of their analytical applications (Cimerman *et al.*,  1997). They are also important ligands, which have been reported to have mild bacteriostatic activity and are used as potential oral iron-chelating drugs for genetic disorders such as thalassemia (Offe *et al.*, 1952; Richardson *et al.*,  1988). Metal complexes based on Schiff bases have received considerable attention because they can be utilized as model compounds of active centres in various complexes (Tamboura *et al.*, 2009). We report here the crystal structure of the title compound (Fig. 1).

The acetohydrazide group is planar and it forms a dihedral angle of 19.7 (1)° with the benzene ring. One of the methoxy groups is coplanar with the attached benzene ring [C1—O1—C4—C5 = -1.7 (3)°] whereas the other is slightly twisted [C2—O2—C3—C8 = 6.3 (3)°]. The molecule adopts a *trans* configuration with respect to the C═N bond. Bond lengths and angles are comparable to those observed for *N*'-[1-(4-methoxyphenyl)ethylidene]acetohydrazide (Li *et al.*, 2008).

The molecules are linked into a chain along the *a* axis by N—H···O hydrogen bonds (Table 1). The chains are cross-linked into a three-dimensional network by C—H···O hydrogen bonds (Fig.2).

### Experimental

3,4-Dimethoxybenzaldehyde (1.66 g, 0.01 mol) and acetohydrazide (0.74 g, 0.01 mol) were dissolved in stirred methanol (25 ml) and left for 2.5 h at room temperature. The resulting solid was filtered off and recrystallized from ethanol to give the title compound in 90% yield. Single crystals suitable for X-ray analysis were obtained by slow evaporation of an ethanol solution at room temperature (m.p. 470–472 K).

### Refinement

H atoms were positioned geometrically (N-H =  0.86 Å and C-H = 0.93 or 0.96Å) and refined using a riding model, with *U*_iso_(H) = 1.2*U*_eq_(C,N) and 1.5*U*_eq_(C_methyl_). A rotating group model was used for the methyl groups.

### Crystal data

**Table:**

C_11_H_14_N_2_O_3_	*F*(000) = 944
*M**_r_* = 222.24	*D*_x_ = 1.259 Mg m^−^^3^
Orthorhombic, *P**b**c**a*	Mo *K*α radiation, λ = 0.71073 Å
Hall symbol: -P 2ac 2ab	Cell parameters from 2070 reflections
*a* = 8.794 (3) Å	θ = 1.7–25.0°
*b* = 10.920 (3) Å	µ = 0.09 mm^−^^1^
*c* = 24.418 (7) Å	*T* = 223 K
*V* = 2345.0 (12) Å^3^	Block, colourless
*Z* = 8	0.24 × 0.21 × 0.20 mm

### Data collection

**Table:**

Bruker SMART CCD area-detector diffractometer	2070 independent reflections
Radiation source: fine-focus sealed tube	1819 reflections with *I* > 2σ(*I*)
graphite	*R*_int_ = 0.034
φ and ω scans	θ_max_ = 25.0°, θ_min_ = 1.7°
Absorption correction: multi-scan (*SADABS*; Bruker, 2002)	*h* = −10→10
*T*_min_ = 0.977, *T*_max_ = 0.979	*k* = −12→12
11332 measured reflections	*l* = −27→28

### Refinement

**Table:**

Refinement on *F*^2^	Secondary atom site location: difference Fourier map
Least-squares matrix: full	Hydrogen site location: inferred from neighbouring sites
*R*[*F*^2^ > 2σ(*F*^2^)] = 0.050	H-atom parameters constrained
*wR*(*F*^2^) = 0.133	*w* = 1/[σ^2^(*F*_o_^2^) + (0.0593*P*)^2^ + 0.6946*P*] where *P* = (*F*_o_^2^ + 2*F*_c_^2^)/3
*S* = 1.10	(Δ/σ)_max_ = 0.001
2070 reflections	Δρ_max_ = 0.15 e Å^−^^3^
149 parameters	Δρ_min_ = −0.12 e Å^−^^3^
0 restraints	Extinction correction: *SHELXL97* (Sheldrick, 2008), Fc^*^=kFc[1+0.001xFc^2^λ^3^/sin(2θ)]^-1/4^
Primary atom site location: structure-invariant direct methods	Extinction coefficient: 0.0113 (16)

### Special details

**Table:**

Geometry. All esds (except the esd in the dihedral angle between two l.s. planes) are estimated using the full covariance matrix. The cell esds are taken into account individually in the estimation of esds in distances, angles and torsion angles; correlations between esds in cell parameters are only used when they are defined by crystal symmetry. An approximate (isotropic) treatment of cell esds is used for estimating esds involving l.s. planes.
Refinement. Refinement of F^2^ against ALL reflections. The weighted R-factor wR and goodness of fit S are based on F^2^, conventional R-factors R are based on F, with F set to zero for negative F^2^. The threshold expression of F^2^ > 2sigma(F^2^) is used only for calculating R-factors(gt) etc. and is not relevant to the choice of reflections for refinement. R-factors based on F^2^ are statistically about twice as large as those based on F, and R- factors based on ALL data will be even larger.

### Fractional atomic coordinates and isotropic or equivalent isotropic displacement parameters (Å^2^)

**Table:**

	*x*	*y*	*z*	*U*_iso_*/*U*_eq_	
O1	−0.00337 (17)	0.67057 (13)	0.22113 (6)	0.0725 (5)	
O2	−0.21387 (16)	0.81158 (15)	0.18477 (6)	0.0738 (5)	
O3	−0.07522 (17)	1.32783 (14)	0.01901 (7)	0.0735 (5)	
C3	−0.0722 (2)	0.83356 (17)	0.16379 (7)	0.0516 (5)	
C7	0.1114 (2)	0.93669 (17)	0.10716 (7)	0.0519 (5)	
C4	0.0432 (2)	0.75598 (17)	0.18390 (8)	0.0537 (5)	
C6	0.2227 (2)	0.8587 (2)	0.12649 (8)	0.0621 (5)	
H6	0.3216	0.8663	0.1135	0.074*	
C8	−0.0381 (2)	0.92289 (17)	0.12603 (7)	0.0507 (5)	
H8	−0.1143	0.9742	0.1130	0.061*	
C10	0.0460 (2)	1.29542 (18)	−0.00180 (8)	0.0556 (5)	
C5	0.1896 (2)	0.76932 (19)	0.16500 (9)	0.0625 (6)	
H5	0.2663	0.7184	0.1780	0.075*	
C9	0.1527 (2)	1.03131 (19)	0.06757 (8)	0.0564 (5)	
H9	0.2501	1.0303	0.0528	0.068*	
C11	0.1217 (3)	1.3670 (2)	−0.04662 (9)	0.0702 (6)	
H11A	0.0706	1.3517	−0.0807	0.105*	
H11B	0.2261	1.3423	−0.0496	0.105*	
H11C	0.1169	1.4528	−0.0382	0.105*	
C2	−0.3390 (2)	0.8771 (3)	0.16274 (10)	0.0825 (7)	
H2A	−0.3234	0.9633	0.1680	0.124*	
H2B	−0.4307	0.8524	0.1810	0.124*	
H2C	−0.3476	0.8600	0.1243	0.124*	
C1	0.1076 (3)	0.5863 (2)	0.24102 (11)	0.0827 (8)	
H1A	0.1518	0.5429	0.2108	0.124*	
H1B	0.0601	0.5291	0.2655	0.124*	
H1C	0.1857	0.6303	0.2602	0.124*	
N2	0.12042 (19)	1.19408 (15)	0.01359 (6)	0.0569 (4)	
H2	0.2071	1.1778	−0.0010	0.068*	
N1	0.06138 (18)	1.11504 (14)	0.05240 (6)	0.0544 (4)	

### Atomic displacement parameters (Å^2^)

**Table:**

	*U*^11^	*U*^22^	*U*^33^	*U*^12^	*U*^13^	*U*^23^
O1	0.0779 (10)	0.0661 (9)	0.0736 (10)	−0.0030 (7)	−0.0097 (8)	0.0243 (8)
O2	0.0572 (9)	0.0863 (11)	0.0779 (10)	0.0039 (8)	0.0052 (7)	0.0262 (8)
O3	0.0634 (9)	0.0642 (10)	0.0929 (11)	0.0057 (7)	−0.0005 (8)	0.0069 (8)
C3	0.0533 (11)	0.0536 (11)	0.0479 (10)	−0.0003 (9)	−0.0020 (8)	−0.0001 (8)
C7	0.0581 (11)	0.0515 (11)	0.0462 (10)	0.0034 (9)	−0.0006 (8)	−0.0017 (8)
C4	0.0650 (12)	0.0477 (10)	0.0486 (10)	−0.0021 (9)	−0.0085 (9)	0.0030 (8)
C6	0.0545 (11)	0.0661 (13)	0.0656 (12)	0.0041 (10)	0.0016 (10)	0.0063 (10)
C8	0.0560 (11)	0.0488 (10)	0.0473 (10)	0.0068 (8)	−0.0032 (8)	0.0010 (8)
C10	0.0587 (12)	0.0505 (11)	0.0576 (11)	−0.0049 (9)	−0.0092 (9)	−0.0037 (9)
C5	0.0624 (12)	0.0574 (12)	0.0679 (13)	0.0097 (10)	−0.0096 (10)	0.0071 (10)
C9	0.0559 (11)	0.0605 (12)	0.0529 (10)	0.0042 (10)	0.0022 (9)	0.0027 (9)
C11	0.0918 (16)	0.0530 (12)	0.0659 (13)	−0.0018 (11)	−0.0030 (12)	0.0071 (10)
C2	0.0548 (12)	0.1042 (19)	0.0886 (17)	0.0095 (13)	0.0055 (12)	0.0167 (15)
C1	0.0994 (18)	0.0651 (14)	0.0836 (16)	0.0000 (13)	−0.0244 (14)	0.0224 (13)
N2	0.0577 (9)	0.0551 (10)	0.0578 (9)	0.0008 (8)	0.0040 (8)	0.0078 (8)
N1	0.0572 (9)	0.0525 (9)	0.0536 (9)	−0.0042 (8)	0.0005 (7)	0.0052 (7)

### Geometric parameters (Å, °)

**Table:**

O1—C4	1.365 (2)	C10—C11	1.501 (3)
O1—C1	1.427 (3)	C5—H5	0.93
O2—C3	1.368 (2)	C9—N1	1.272 (2)
O2—C2	1.419 (3)	C9—H9	0.93
O3—C10	1.233 (2)	C11—H11A	0.96
C3—C8	1.375 (3)	C11—H11B	0.96
C3—C4	1.410 (3)	C11—H11C	0.96
C7—C6	1.381 (3)	C2—H2A	0.96
C7—C8	1.401 (3)	C2—H2B	0.96
C7—C9	1.461 (3)	C2—H2C	0.96
C4—C5	1.375 (3)	C1—H1A	0.96
C6—C5	1.386 (3)	C1—H1B	0.96
C6—H6	0.93	C1—H1C	0.96
C8—H8	0.93	N2—N1	1.383 (2)
C10—N2	1.340 (2)	N2—H2	0.86
			
C4—O1—C1	117.52 (18)	N1—C9—H9	118.5
C3—O2—C2	118.41 (16)	C7—C9—H9	118.5
O2—C3—C8	125.05 (17)	C10—C11—H11A	109.5
O2—C3—C4	114.81 (17)	C10—C11—H11B	109.5
C8—C3—C4	120.14 (18)	H11A—C11—H11B	109.5
C6—C7—C8	119.11 (18)	C10—C11—H11C	109.5
C6—C7—C9	119.09 (18)	H11A—C11—H11C	109.5
C8—C7—C9	121.80 (17)	H11B—C11—H11C	109.5
O1—C4—C5	125.20 (18)	O2—C2—H2A	109.5
O1—C4—C3	115.24 (18)	O2—C2—H2B	109.5
C5—C4—C3	119.55 (18)	H2A—C2—H2B	109.5
C7—C6—C5	121.14 (19)	O2—C2—H2C	109.5
C7—C6—H6	119.4	H2A—C2—H2C	109.5
C5—C6—H6	119.4	H2B—C2—H2C	109.5
C3—C8—C7	120.12 (17)	O1—C1—H1A	109.5
C3—C8—H8	119.9	O1—C1—H1B	109.5
C7—C8—H8	119.9	H1A—C1—H1B	109.5
O3—C10—N2	122.96 (19)	O1—C1—H1C	109.5
O3—C10—C11	122.33 (19)	H1A—C1—H1C	109.5
N2—C10—C11	114.71 (19)	H1B—C1—H1C	109.5
C4—C5—C6	119.93 (19)	C10—N2—N1	121.62 (17)
C4—C5—H5	120.0	C10—N2—H2	119.2
C6—C5—H5	120.0	N1—N2—H2	119.2
N1—C9—C7	122.97 (18)	C9—N1—N2	114.28 (17)
			
C2—O2—C3—C8	6.3 (3)	C6—C7—C8—C3	0.7 (3)
C2—O2—C3—C4	−173.8 (2)	C9—C7—C8—C3	−179.28 (17)
C1—O1—C4—C5	−1.7 (3)	O1—C4—C5—C6	179.13 (18)
C1—O1—C4—C3	177.38 (18)	C3—C4—C5—C6	0.1 (3)
O2—C3—C4—O1	0.1 (2)	C7—C6—C5—C4	1.1 (3)
C8—C3—C4—O1	−179.94 (16)	C6—C7—C9—N1	−171.95 (19)
O2—C3—C4—C5	179.25 (18)	C8—C7—C9—N1	8.1 (3)
C8—C3—C4—C5	−0.8 (3)	O3—C10—N2—N1	3.4 (3)
C8—C7—C6—C5	−1.5 (3)	C11—C10—N2—N1	−176.62 (17)
C9—C7—C6—C5	178.54 (18)	C7—C9—N1—N2	−178.40 (17)
O2—C3—C8—C7	−179.67 (18)	C10—N2—N1—C9	−171.88 (17)
C4—C3—C8—C7	0.4 (3)		

### Hydrogen-bond geometry (Å, °)

**Table:**

*D*—H···*A*	*D*—H	H···*A*	*D*···*A*	*D*—H···*A*
N2—H2···O3^i^	0.86	1.97	2.803 (2)	164
C1—H1C···O2^ii^	0.96	2.55	3.435 (3)	153
C11—H11C···O3^iii^	0.96	2.47	3.425 (3)	174

Symmetry codes: (i) *x*+1/2, −*y*+5/2, −*z*; (ii) *x*+1/2, *y*, −*z*+1/2; (iii) −*x*, −*y*+3, −*z*.
